# Memory performance-related dynamic brain connectivity indicates pathological burden and genetic risk for Alzheimer’s disease

**DOI:** 10.1186/s13195-017-0249-7

**Published:** 2017-03-31

**Authors:** Frances C. Quevenco, Maria G. Preti, Jiri M. G. van Bergen, Jun Hua, Michael Wyss, Xu Li, Simon J. Schreiner, Stefanie C. Steininger, Rafael Meyer, Irene B. Meier, Adam M. Brickman, Sandra E. Leh, Anton F. Gietl, Alfred Buck, Roger M. Nitsch, Klaas P. Pruessmann, Peter C. M. van Zijl, Christoph Hock, Dimitri Van De Ville, Paul G. Unschuld

**Affiliations:** 1 Institute for Regenerative Medicine (IREM), University of Zurich, Zurich, Switzerland; 2 Hospital for Psychogeriatric Medicine, University of Zurich, Minervastr.145, CH-8032 Zurich, Switzerland; 3 Institute for Biomedical Engineering, University of Zurich and ETH Zurich, Zurich, Switzerland; 4 Division of Nuclear Medicine, University of Zurich, Zurich, Switzerland; 5 Department of Radiology, Johns Hopkins School of Medicine and F.M. Kirby Center for Functional Brain Imaging at Kennedy Krieger Institute, Baltimore, MD USA; 6 Department of Radiology and Medical Informatics, Université de Genève, Geneva, Switzerland; 7 Institute of Bioengineering, École polytechnique fédérale de Lausanne, Lausanne, Switzerland; 8 Taub Institute for Research on Alzheimer’s Disease and the Aging Brain, Department of Neurology, College of Physicians and Surgeons, Columbia University, New York, USA

**Keywords:** Alzheimer’s disease, Amyloid beta, Iron, Episodic memory, Oxidative stress, Dynamic functional connectivity

## Abstract

**Background:**

The incidence of Alzheimer’s disease (AD) strongly relates to advanced age and progressive deposition of cerebral amyloid-beta (Aβ), hyperphosphorylated tau, and iron. The purpose of this study was to investigate the relationship between cerebral dynamic functional connectivity and variability of long-term cognitive performance in healthy, elderly subjects, allowing for local pathology and genetic risk.

**Methods:**

Thirty seven participants (mean (SD) age 74 (6.0) years, Mini-Mental State Examination 29.0 (1.2)) were dichotomized based on repeated neuropsychological test performance within 2 years. Cerebral Aβ was measured by ^11^C Pittsburgh Compound-B positron emission tomography, and iron by quantitative susceptibility mapping magnetic resonance imaging (MRI) at an ultra-high field strength of 7 Tesla (7T). Dynamic functional connectivity patterns were investigated by resting-state functional MRI at 7T and tested for interactive effects with genetic AD risk (apolipoprotein E (ApoE)-ε4 carrier status).

**Results:**

A relationship between low episodic memory and a lower expression of anterior-posterior connectivity was seen (F(9,27) = 3.23, *p* < 0.008), moderated by ApoE-ε4 (F(9,27) = 2.22, *p* < 0.005). Inherent node-strength was related to local iron (F(5,30) = 13.2; *p* < 0.022).

**Conclusion:**

Our data indicate that altered dynamic anterior-posterior brain connectivity is a characteristic of low memory performance in the subclinical range and genetic risk for AD in the elderly. As the observed altered brain network properties are associated with increased local iron, our findings may reflect secondary neuronal changes due to pathologic processes including oxidative stress.

## Background

Sporadic Alzheimer’s disease (AD) is the most frequent cause of dementia [1] and is characterized pathologically by deposition of amyloid-beta (Aβ), hyperphosphorylated tau, and progressive neuronal dysfunction [2, 3]. Pathological brain change in AD furthermore includes the increased accumulation of cerebral iron, which has been linked to several pathological processes associated with risk for AD and disease progression [4–9].

Clinically, AD develops gradually and presents with progressive decline in multiple cognitive domains, particularly affecting episodic memory, executive functioning, and perceptual speed [10–13]. While these alterations may also take place during normal aging, the concurrent incidence of subtle cognitive dysfunction and emerging AD brain pathology in the cognitively normal elderly is considered to reflect a preclinical stage of AD [14, 15]. Moreover, progression of AD is significantly affected by genetic predisposition and presence of the apolipoprotein E (ApoE) ε4 allele, which is the strongest known genetic risk factor for late-onset AD [16–20].

A variety of noninvasive neuroimaging techniques have been developed to assess AD pathology directly and AD-associated neurobiological changes. Positron emission tomography (PET) using the ^11^C-labeled tracer Pittsburgh Compound-B (PiB), is a validated method for measuring fibrillar Aβ [21, 22] and has been used to assess pathological burden in several clinical studies [23–25].

Cerebral iron can be measured in vivo by applying quantitative susceptibility mapping (QSM) magnetic resonance imaging (MRI) [26, 27], which has very high signal-to-noise ratios (SNRs) when acquired at ultra-high field strength [28, 29].

While the blood-oxygen-level dependent (BOLD) functional MRI (fMRI) contrast reflects in vivo neuronal activity [30, 31], an ultra-high field strength of 7 Tesla (7T) may provide enhanced SNR and enriched contrast [32–35]. Therefore, for the current study, a three-dimensional T2-weighted BOLD fMRI sequence was used (“T2-prep fMRI”), which was specifically designed for ultra-high magnetic field strength MRI acquisition [36]. Functional connectivity, as inferred from synchronous fluctuations in activity in spatially distant brain areas [37], is an established measure for investigating the integrity of functional brain networks and their potential impairment in AD [38–41]. Investigation of “dynamic functional connectivity” additionally provides information on the expression of brain networks over time and has been used to characterize changes in brain network connectivity in neuropsychiatric disorders earlier [42–45].

The primary aim of this study was to investigate whether dynamic expression of cognitive brain networks relates to interindividual variation of cognitive performance in healthy elderly subjects and their genetic predisposition for AD. Considering that “stationary” connectivity is significantly affected by local neurodegenerative brain change [46–50], the second aim of this study was to examine the relationship between dynamic network expression and neuropathology. ^11^C-PiB-PET and QSM-MRI were thus used for measuring neuropathological burden, as indicated by the accumulation of cerebral Aβ and iron. QSM-MRI and resting state T2-prep fMRI were performed at an ultra-high field strength of 7T. Interindividual variability of cognitive performance over time was assessed by performing two neuropsychological tests for each participant, 2 years apart.

## Methods

### Study population

The study sample included 37 (13 females and 24 males) cognitively normal Swiss-German elderly adults (mean age (SD) 73 (6.6) years, range 62–89 years, mean education (SD) 14 (3) years, range 8–20) living in the canton Zurich from an ongoing study [51], who received ultra-high field strength MRI at 7T and neuropsychological follow-up after 2 years (Table 1). Genotyping of the APOE gene (rs429358 and rs7412) revealed 13 carriers with at least one ApoE-ε4 allele (two subjects had the genotype ε4/ε4). All study procedures were carried out in concordance with regulations issued by the local ethics authority (Kantonale Ethikkommission Zürich, www.kek.zh.ch), as well as good clinical practice (GCP) guidelines and the declaration of Helsinki [52]. Written informed consent was obtained from all participants before inclusion in the study. Inclusion criteria were age between 55 and 80 years, no significant cognitive impairment as indicated by Mini-Mental State Examination (MMSE) <26, no acute medical or neurological comorbidities, and no present psychiatric disorder or current substance abuse. Exclusion criteria included evidence of infarction and focal or significant hemorrhagic lesions in the MRI, as indicated in detail previously for neuroimaging studies on nondemented elderly populations at our center [8, 41, 51, 53].

**Table 1:** Mean scores of neuropsychological tests at inclusion of study and follow-up, as well as changes (%) in performance between sessions

Test	Inclusion	Follow-up	*p* value^*^	Total sample (*n* = 37), % change per year	No decline, % change per year	No decline *p* value^*^	Decline, % change per year	Decline *p* value^*^
MMSE	29.03 (1.17)	29.03 (1.19)	1.00	0.00	1.83% (*n* = 23)	<0.001	–1.97% (*n* = 14)	<0.0001
BNT	14.32 (1)	14.46 (0.84)	0.53	0.01	1.45% (*n* = 33)	<0.01	–5.53% (*n* = 4)	<0.015
DSF	6.81 (1.66)	6.84 (1.55)	0.94	0.02	10.32% (*n* = 24)	<0.01	–14.09% (*n* = 13)	<0.001
DSB	6.08 (1.57)	5.95 (1.73)	0.73	0.00	10.32% (*n* = 20)	<0.001	–11.69% (*n* = 17)	<0.0001
TMT B/A	2.83 (1.27)	2.61 (1)	0.41	0.00	17.51% (*n* = 19)	<0.002	–18.84% (*n* = 18)	<0.001
VLMT delayed recall	8.49 (3.92)	9.62 (4.21)	0.23	0.19	46.22% (*n* = 19)	<0.001	–9.31% (*n* = 18)	<0.01

Values are shown as mean (SD)

^*^Follow-up versus inclusion

*BNT* Boston Naming Test, *DSB* Digit Span Backward, *DSF* Digit Span Forward, *MMSE* Mini-Mental State Examination, *TMT B/A* Trail Making Test part B/part A, *VLMT* Verbal Learning and Memory Test

### Cognitive assessment of participants

All subjects were evaluated by neuropsychological tests as described previously [51]. For the current study, neuropsychological follow-up was scheduled after 2 years and included German language versions of the MMSE as a general measure of cognitive performance [54], as well as domain-specific testing using the Digit Span Forward (DSF) and Digit Span Backward (DSB) for measuring working memory [55], the delayed recall Verbal Learning and Memory Test (VLMT) [56] for assessing episodic memory, the abbreviated CERAD Boston Naming Test (BNT) on confrontational word retrieval [57, 58], and the ratio between part B and A of the Trail Making Test (TMT) as a measure of executive function [59]. To characterize the performance of each measure over time, the percentage difference between follow-up value and baseline was used to obtain yearly variability ratios (relative change per 365 days) for each participant and each investigated cognitive domain. Subjects were allocated to the group “Decline” if the respective yearly variability ratios were negative and to the group “No Decline” if ratios were equal or better than at inclusion (Table 1).

### Acquisition of MRI data

Participants underwent one session of scanning using a Philips 7-Tesla Achieva whole-body scanner (Philips Healthcare, Best, The Netherlands) equipped with a Nova Medical quadrature transmit head coil and 32-channel receive coil array, located at the Swiss Federal Institute of Technology (ETH) in Zurich, as reported previously [8, 41, 53]. For the current study, this scanner was used to acquire T1-weighted MP2RAGE image [60] data (TR/TE = 4.8 ms/2.1 ms, voxel size = 0.6 × 0.6 × 0.6 mm^3^, SENSE-factor = 2 × 1 × 2, scan duration = 7:50 min) for referencing and automated image segmentation. Resting-state fMRI data was acquired using a 3D T2-prep gradient recalled echo (GRE) sequence, optimized for ultra-high field strength acquisition [36] (TR = 2 s, TR_GRE_/TE_GRE_ = 3.08 ms/1.6 ms, voxel size = 1.5 × 1.5 × 1.5 mm^3^, scan duration = 7:03 min). Iron load was measured by QSM-MRI at ultra-high field strength using a multi-echo three-dimensional gradient recalled echo (GRE) sequence with three echoes (TR/TE/ΔTE = 23/6/6 ms, flip angle = 10°, voxel size = 0.5 × 0.5 × 0.5 mm^3^, SENSE-factor = 2.5 × 1 × 2, flow-compensated, scan duration = 13:48 min) for acquiring anatomical MR measures of magnitude and phase for calculation of QSM images using a previously described pipeline [8, 29].

### Acquisition of PET data

Cerebral Aβ was measured with ^11^C-PiB-PET [8, 41, 53]. Briefly, participants were administered a dose of 350 MBq of the ^11^C-labeled tracer intravenously and cerebral amyloid deposition was estimated based on late frame signals representing 50–70 min. Measures of individual brain Aβ load were derived from the ratio of standardized uptake values (SUV) of regional PiB referenced to cerebellar SUV after coregistration using the PMOD brain tool (PNEURO) software, Version 3.4 (PMOD Technologies Ltd., Zurich, Switzerland).

### Processing of fMRI data

Structural and functional images were preprocessed with a standardized in-house-developed preprocessing pipeline [61] implemented in MATLAB scripts (MATLAB 2015b, Version 8.6; MathWorks Inc., Natick, MA, USA), which included functions from SPM8, SPM12 (http://www.fil.ion.ucl.ac.uk/spm/) and DPARSFA toolboxes [62]. Images were spatially realigned and smoothed (FWHM = 5 mm). Nuisance variables were regressed out from the regional time series (these included linear and quadratic trends, six head motion parameters, average cerebrospinal fluid signal from ventricular masks and white matter signal from white matter masks). All the functional data was postprocessed using the Artifact Detection Tool (ART; www.nitrc.org/projects/artifact_detect/), resulting in exclusion of three subjects from further analysis due to excess motion (>2 mm). The remaining 37 functional volumes were regionally parcellated using the automated anatomical labeling (AAL) atlas [63], resulting in the definition of 90 anatomical grey-matter regions of interest. Regional mean time series were then extracted by averaging the preprocessed BOLD signal over all voxels in each region and filtered with a band-pass filter cutoff (0.017–0.15 Hz), as performed previously for investigating dynamic functional networks at rest [45].

### Assessment of dynamic functional connectivity

A whole brain dynamic functional connectivity (FC) matrix for each subject was calculated using a sliding time window approach (window size = 30 TRs = 60 s; step =1 TR = 2s) to calculate the time-varying correlations between BOLD fluctuations in distinct brain regions, as reported previously [45, 64]. Briefly, in order to account for spurious fluctuations while preserving meaningful fluctuations within the time windows, the window size was determined by calculating 1/smallest frequency in the data, represented by the high-pass filter cutoff [64]. The FC matrices obtained for all windows were then vectorized and temporally concatenated to obtain a dFC (connections × time) matrix, which was normalized by first removing the global mean and dividing for the global standard deviation and secondly applying a row-wise demeaning to focus on the exclusive contributions of dynamics. Finally, dFC data were concatenated into a (connections × time*subjects) matrix and principal component analysis (PCA) was used for reducing data dimensionality and to define subject specific eigenconnectivities, referring to distinct connectivity patterns [45] (i.e., building blocks of dFC, highlighting dominant patterns of FC increase/decrease that recur across time and in the population). For the current study, the following properties of dynamic network connectivity were investigated: 1) Time-contributed weights and percentage of positive weights were obtained for identified connectivity patterns as a measure of dynamic FC changes and compared between groups for inferring expression of connectivity patterns within the acquired fMRI data (“network expression”). Effects of cognitive decline and ApoE-ε4 status on changes in dynamic FC network expression were assessed using a multivariate Hotelling’s T2 test. Singular value decomposition with bootstrapping matched to a Procrustes transform [65] was conducted to test the reproducibility of the eigenconnectivity patterns [66]. Through this process, the stronger connections are attributed higher values and the weaker connections are gradually discarded. This eases interpretation without affecting group comparison tests and these data were therefore used for the visualization of the eigenconnectivity networks. 2.) Node strengths for significant connectivity patterns were calculated as a graph theoretical measure indicating involvement of a particular region in a network, as derived from the sum of all connection weights for all connections attached to a given node [67].

### Statistical analysis

All data processing was performed in MATLAB (2015b, Version 8.6) and its Statistics and Machine Learning Toolbox (Version 10.0). A Hotelling’s T2 multivariate test in combination with sequentially rejective Holm-Bonferroni correction [68] for investigating variability indices pertaining to six neuropsychological tests was used to assess relationship with expression of dynamic connectivity patterns. To follow-up on significant associations, secondary analysis investigated: 1) interactive effects of test performance and ApoE-ε4 on network alterations using a multivariate Hotelling’s T2 test; and 2) relationships between node strength of significant connectivity patterns and local Aβ and iron load, respectively, by applying false discovery rate (FDR)-corrected permutation-based multivariate analysis of variance (MANOVA) [69]. Effect sizes of differences between the “Decline” and “No decline” groups were estimated using Cohen’s *d* [70].

## Results

### Stratification of the study sample by identification of participants with lower cognitive performance after 2 years

**Fig. 1:**
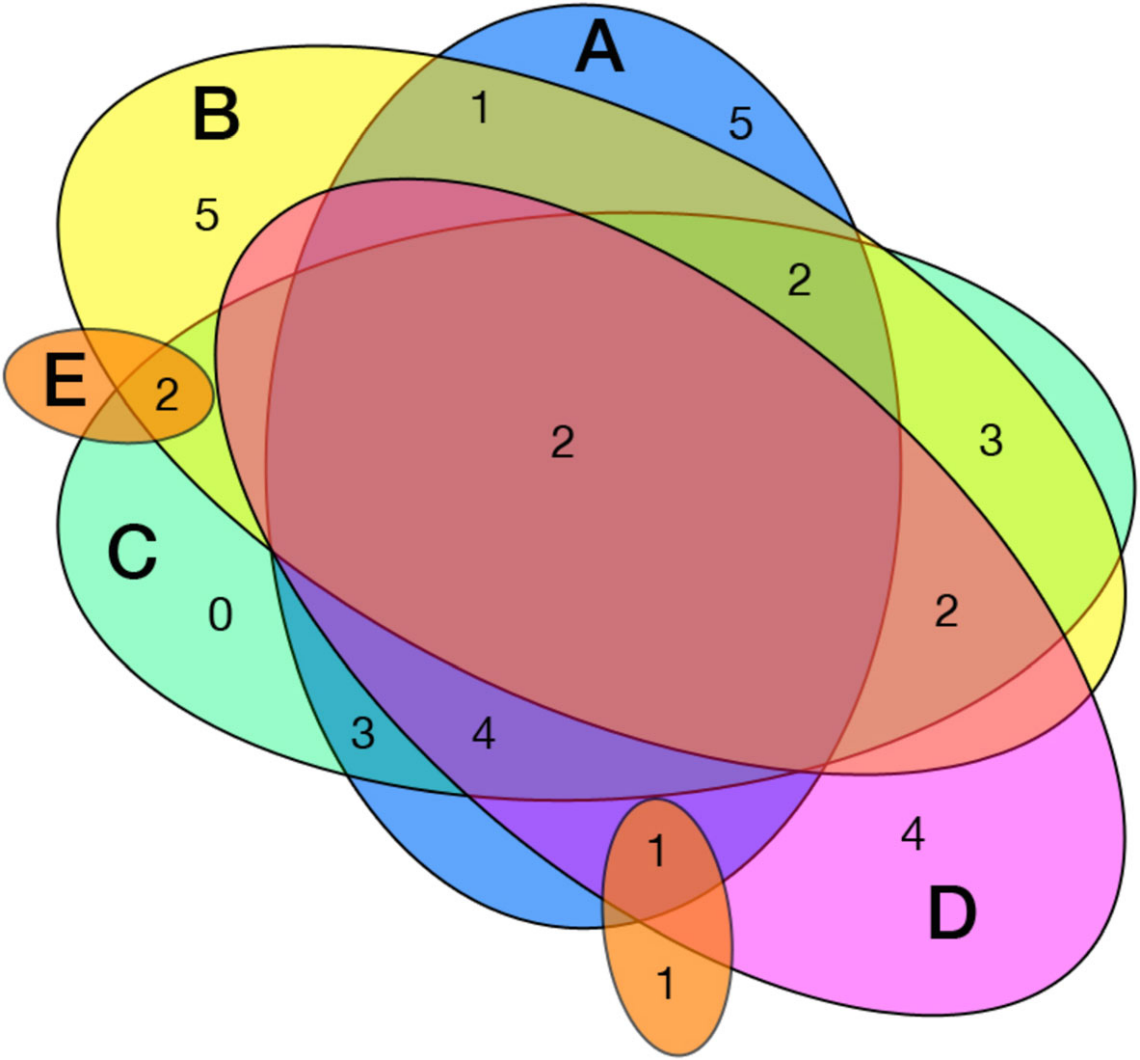
Venn diagram illustrating the relationship of “decliners” among the different strata. Each circle is labeled according to the neuropsychological test it represents: A, delayed recall Verbal Learning and Memory Test; B, Digit Span Backward test; C, Trail-Making Test; D, Digit Span Forward test; E, Boston Naming Test). The *small circle* without a label in the same color as E also illustrates decliners in the Boston Naming Test

Participants were neuropsychologically tested at baseline and after 2 years (mean (SD) follow-up time between testing, 719 (277) days). All participants had normal levels of general cognitive performance both at inclusion (mean (SD) MMSE 29.03 (1.17)), as well as at follow-up (mean (SD) MMSE 29.03 (1.19), mean average change per year 0.39%) and for the entire population (*n* = 37) no significant difference between baseline and follow-up could be observed for the investigated neuropsychological tests. For stratifying the study population by cognitive performance over time, a subgroup of “decliners” was defined that included subjects with negative yearly variability ratios for each cognitive domain. Decliners thus included four subjects for the Boston Naming Test (average yearly change –5.53%), 13 subjects for the Digit Span Forward Test (average yearly change –14.09%), 17 subjects for the Digit Span Backward Test (average yearly change –11.69%), 18 subjects for Trail-Making Test ratio (average yearly change –18.84%), and 18 subjects for the VLMT delayed recall test (average yearly change –9.31%) (Table 1). By generating a Venn diagram, a small degree of overlap between decline in the respective domains could be visualized (Fig. 1), indicating no participant with concurrent decline in all five tests and only two subjects with declined performance in four out of five tests. Moreover, the null hypothesis that the performed tests for domain performance assessed one common factor was dismissed by means of a factor analysis (χ^2^(5) = 11.995, *p* < 0.04).

### Lower expression of an anterior-posterior network in subjects with declined episodic memory performance

**Fig. 2:**
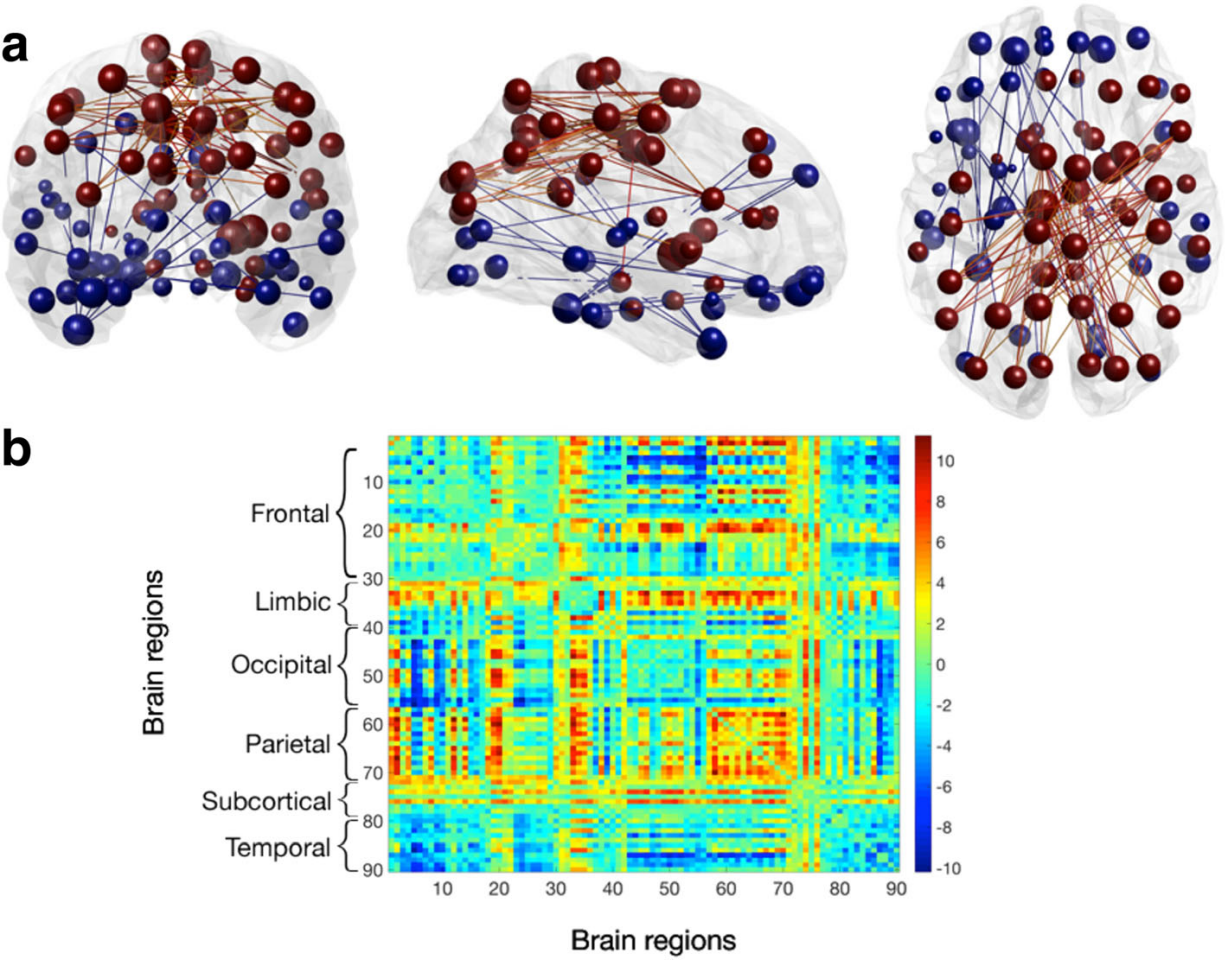
**a** Coronal, sagittal and axial views of the 2% strongest connections in brain space of the anterior-posterior network. Brain regions are shown as spheres where their size represents their degree and color represents the algebraic sign of relative node strength (*red* for positive, *blue* for negative). Connections follow the same color scheme. **b** Corresponding eigenconnectivity for anterior-posterior network. Plot follows an identical color scheme as the glass brains. Each number on the *x* and *y* axes represents a label on the AAL atlas

Individuals characterized as having memory decline showed reduced expression of an anterior-posterior connectivity pattern, which remained significant (alpha = 5%) after applying Holm-Bonferroni correction for multiple testing (Roy’s maximum root F(9,27) = 3.23, *p* < 0.0078; factor loading –0.521; Fig. 2a and b). No significant effects (Roy’s maximum root test, indicated are uncorrected *p* values without adjustment for multiple testing) on dynamic FC were found for groups defined by the cognitive tests Boston Naming (F(9,27) = 0.53, *p* = 0.85), Digit Span Forward (F(9,27) = 1.33, *p* = 0.27), Digit Span Backward (F(9,27) = 0.97, *p* = 0.48) and Trail Making Test B/A (F(9,27) = 0.84, *p* = 0.6).

### Node strength is associated with higher regional iron, but not amyloid-beta

**Fig. 3:**
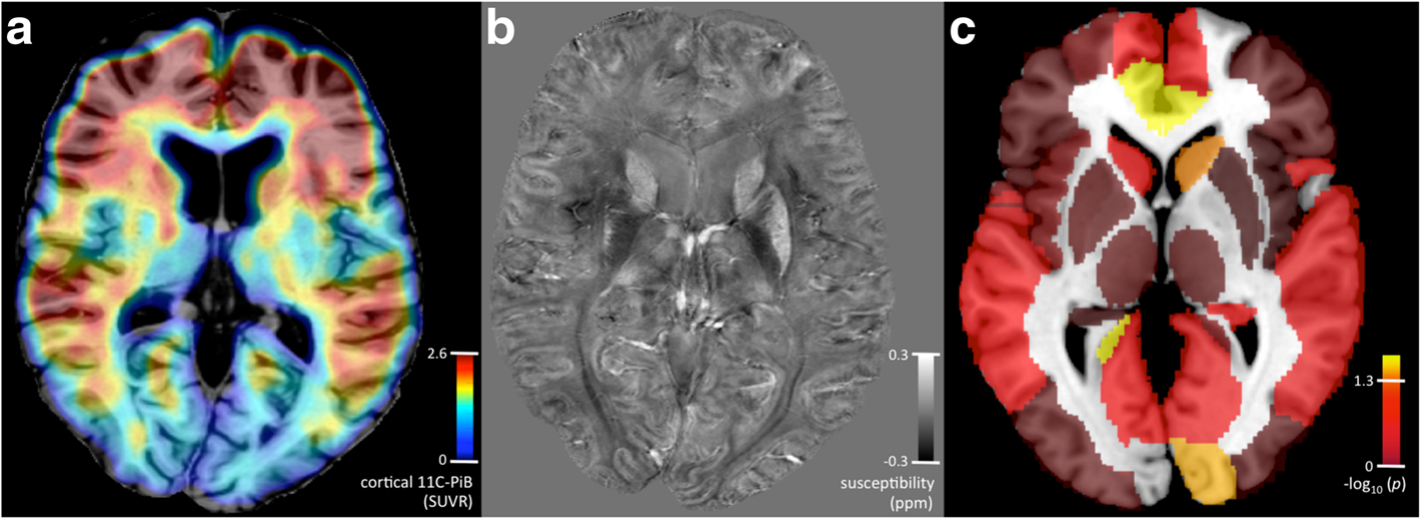
**a** Exemplary ^11^C-PiB-PET image, indicating regional distribution of standardized uptake value ratio (*SUVR*) as a measure of local Aβ deposition. **b** Exemplary QSM image at 7T, indicating regional distribution of susceptibility as used for inferring on local iron load. **c** Correlation between node strength of the anterior-posterior network and local iron load. Significance as indicated by alpha of 5% after correction for multiple testing (FDR) was reached at –log_10_(*p*) = 1.3

**Fig. 4:**
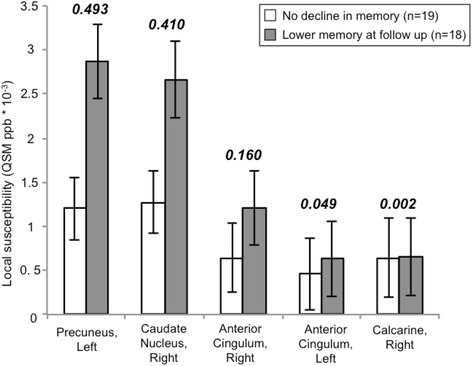
Group differences between subjects with lower memory performance after 2 years and subjects without decline at follow-up, as measured by VLMT delayed recall, in iron load of significant nodes in the anterior-posterior network (MANOVA, *p* < 0.05 after correction for multiple testing by FDR). Indicated is the susceptibility measure, as a quantitative susceptibility mapping (*QSM*)-derived inference on local iron content. Numbers refer to the effect size, as calculated by Cohen’s *d*

The observed relationship between memory decline and anterior-posterior network expression was followed up for potential relationships between node strength of significant connectivity patterns and neuropathology, by permutation MANOVA. This approach yielded a set of five nodes where nodal contributions to the network were associated with significantly higher susceptibility, as an indicator of local iron, in subjects with lower memory performance at follow-up, compared to those with equal or better performance (F(1,35) = 13.1, *p* < 0.022, FDR-corrected; Fig. 3c). The most distinct increases for local iron were observable for the left Precuneus (Cohen’s *d* = 0.493), right Caudate (*d* = 0.410) and right anterior Cingulate (*d* = 0.160) (Fig. 4). While no significant relationship between regional network contribution and local Aβ load could be observed for any of the identified nodes (*p* = 0.128, uncorrected), the association between node strength in the anterior-posterior network and memory decline was not significant when correcting iron levels for Aβ (t(27) = 12.49, *p* = 0.1).

### Combined effects of ApoE-ε4 and declined episodic memory performance on network expression

**Fig. 5:**
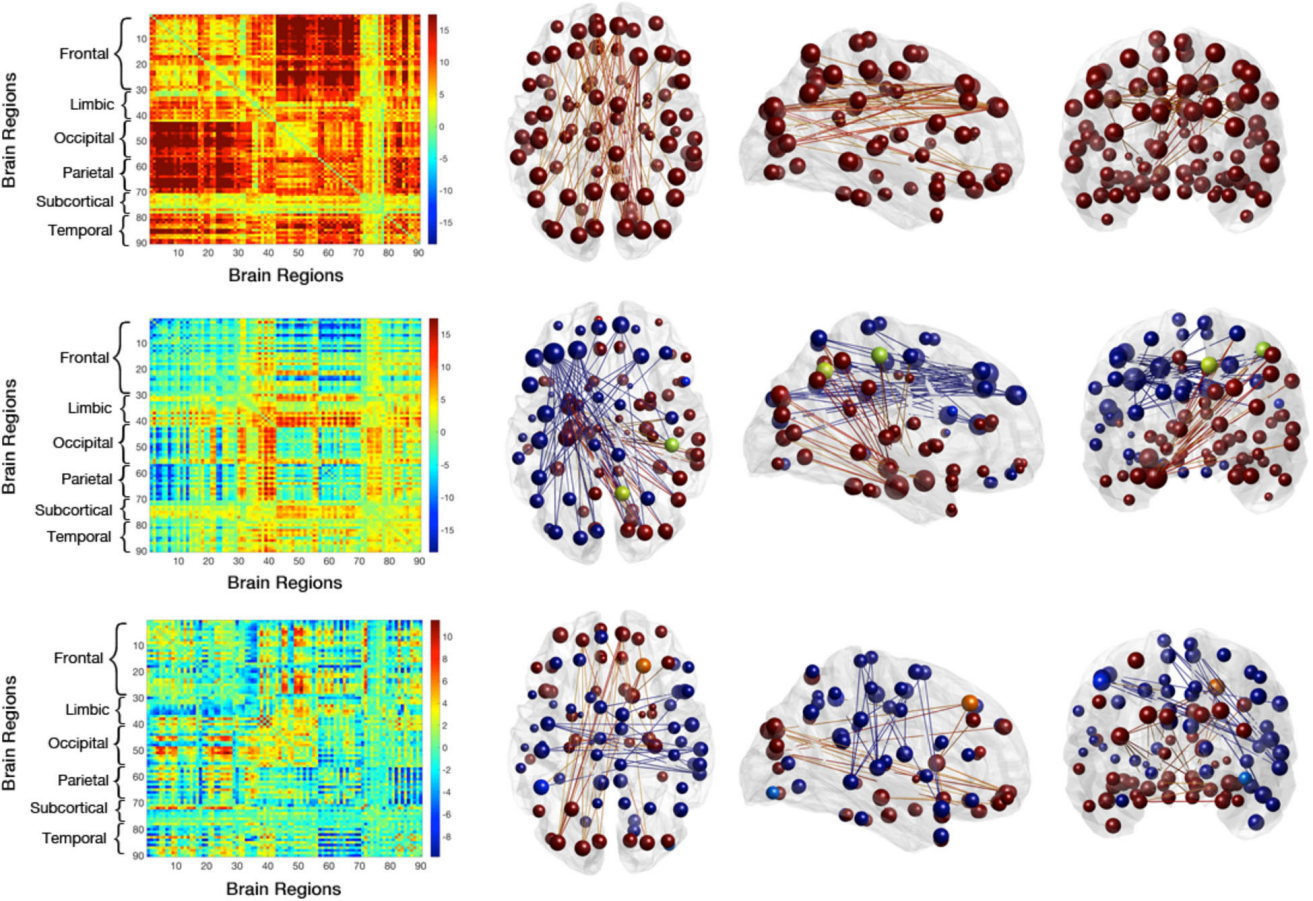
Axial, sagittal and coronal views of the 2% strongest connections in brain space of a global, fronto-temporal, and fronto-occipital network (rows 1–3, respectively), demonstrated to drive the group difference between subjects who display both APOE-e4 expression and memory decline versus the remaining sample. Brain regions are shown as nodes (spheres) where their size represents their degree and the color code matches the algebraic sign of relative node strength (*red* and *yellow* for positive, *green*, *turquoise* and *blue* for negative). Connections follow the same color scheme

A multivariate Hotelling's T2 test was used to investigate whether the observed change of dynamic connectivity in a context of memory decline relates to the individual risk of developing AD. Participants with both ApoE-ε4, as well as declined memory over 2 years (*n* = 6), were tested against the rest of the sample (*n* = 31), resulting in significant differences in the percentage of positive weights between groups (Roy’s maximum root F(9,27) = 2.22, *p* < 0.005). Secondary testing of ApoE-ε4 carriers against noncarriers within the group of subjects with declined memory indicated a nominally significant difference in the percentage of positive weights between groups (Roy’s maximum root F(9,8) = 5.29, *p* < 0.03). Here, the factor loadings of each eigenconnectivity point to three connectivity patterns that drive this group difference: the global network expression (factor loading –0.5), a fronto-temporal network (factor loading –0.44), and a fronto-occipital network (factor loading –0.48). These findings indicate a significant difference in specific network expression associated with ApoE-ε4 carrier status and memory decline, concerning: 1) a pattern showing the alteration of global connectivity (first eigenconnectivity, positive pattern); 2) the alteration between anterior-posterior network connectivity (negative pattern) and interhemispheric fronto-temporal (positive pattern) connectivity (second eigenconnectivity); and 3) the alteration between parieto-temporal connections (negative pattern) and fronto-occipital connectivity (positive pattern) (Fig. 5). The negative sign of the factor loadings indicate that these networks show an increase in the negative patterns and decrease in the positive patterns.

## Discussion

By implementing dynamic functional connectivity analysis on ultra-high field strength MRI at 7T, reduced expression of a dynamic anterior-posterior brain network could be identified as a correlate of low episodic memory performance over time in cognitively normal elderly subjects. While strength of nodes implicated in this network related to mean regional susceptibility as a measure of local iron accumulation, no significant association could be observed for local Aβ plaque density, as inferred by PiB standardized uptake value ratio (SUVR). As dynamic functional connectivity changes relates to both lower episodic memory and ApoE-ε4, our findings may reflect brain change taking place in subjects at increased risk for AD and preclinical stages of AD, respectively. To our knowledge, this is the first study that demonstrates a relationship between memory decline within the normal range and altered dynamic network connectivity as a potential correlate of increased risk for AD in the healthy elderly.

Assessment of pathological burden included estimation of Aβ plaque density by administering ^11^C-PiB-PET, as has been demonstrated to be valid for characterizing progression of AD pathology previously [21, 71–73]. Additionally, iron was measured by QSM-MRI [26, 27] which was performed at ultra-high field strength for maximizing signal quality [28]. While functional connectivity analysis of BOLD-fMRI data is a well-established measure of neural integrity in AD [38, 39, 74], for the current study, T2-prep fMRI was used to avoid signal contortion near air cavities, but which nevertheless benefits from high SNR at 7T [36]. Moreover, dynamic functional connectivity [42] was assessed for inferring on the temporal expression of connectivity patterns by integrating information on both the regional extent and temporal evolution of coherent BOLD activity [45, 64]. The studied population was cognitively assessed by performing tests and follow-up for language capacity, working memory, episodic memory, and executive function within 2 years. While neuropsychological assessment over time has been suggested previously to be a particularly reliable measure of cognitive performance in the elderly [75, 76], the investigated study population in our study remained relatively stable regarding test performance within the study period. However, by splitting the study population by algebraic sign of yearly variability ratios, two subgroups that significantly differed regarding their rate of decline in the investigated cognitive domains could be identified that only showed moderate overlap regarding cognitive domains affected by lower performance over time. Some participants in our study performed better at follow-up, which may be explained by practice effects as reported previously for longitudinal studies on cognitively normal elderly subjects [77].

Our finding of an association between memory performance and dynamic connectivity appears consistent with a concatenation of earlier reports on altered functional connectivity in AD [50, 78, 79] as well as associations between distinct cognitive impairment and increased AD risk [15, 80]. Central nodes of the dynamic anterior-posterior network found to be associated with episodic memory performance exhibited increased iron for the lower episodic memory group. The strongest effects were observable for the left precuneus, right caudate, and right anterior cingulate. This observation appears consistent with previous reports on subcortical regions being primarily affected by iron accumulation in neurodegenerative brain disorders [81, 82]. While no differences in local Aβ plaque density were measured as being associated with network dynamics, a distinct impact of ApoE-ε4 on network expression was observable, suggesting an association with an increased risk for AD. Moreover, cerebral iron accumulation may reflect pathological processes implicated in AD [81, 83–85], and local interactions of accumulated iron have been suggested previously to promote neuronal damage in the context of AD [6, 7, 85–88]. These earlier considerations on interactions between iron and AD pathology may be consistent with our lack of identifying significant associations between local iron and network-dynamics, when iron was corrected for Aβ.

While our data indicate memory decline within the normal range as a potential correlate of altered dynamic network connectivity and increased genetic risk for AD, our findings might furthermore support earlier considerations on the relevance of pathological processes reflected by local iron, such as oxidative stress, free radical activity, and mitochondrial dysfunction [83, 84]. These processes may be reflected by functional changes, primarily affecting brain regions with high metabolic activity and increased susceptibility to age-related damage [89]. Considering these pathological processes as secondary to the earlier manifestation of AD pathology, they nevertheless may substantially contribute to cognitive decline [4, 81, 90, 91] and may thus represent a correlate of the well-established phenomenon of functional disconnection in AD [39, 50, 89]. This interpretation may be consistent with previous considerations on a stronger association of functional impairment with secondary pathological processes than with Aβ plaque density itself [92, 93].

The following limitations have to be allowed for when appraising our reported findings. While neuropsychological performance was assessed based on measures within 2 years, neuroimaging was performed only once and thus only confers cross-sectional information. Additional longitudinal studies are necessary to investigate the temporal relationship of the different constituents of pathological burden, which included Aβ plaque density and iron load in the current study. As the number of study participants affected by cognitive decline, and thus power to identify functional correlates of low performance, varied between the domains investigated, negative findings for language capacity, working memory, and executive function need to be interpreted with caution. Moreover, while MRI at ultra-high field strength may provide advantages in SNR and thus facilitate detection of pathological change [28], reproducibility of findings may be difficult as it requires the implementation of sequences that were originally performed on 7T on more readily available clinical scanners with lower field strength.

## Conclusions

While the association between memory decline in the elderly and emerging AD-related pathology is well established, our findings suggest that variation in the subclinical range of memory performance may be linked to alterations in functional network dynamics. Moreover, our data suggest that altered network dynamics reflect regional pathological burden, as characterized by increased iron accumulation, and also genetic risk, as conferred by ApoE-ε4. Additional studies are necessary to clarify whether the observed dynamic functional changes reflect impaired neural integrity and thus possibly a symptom-free stage of incipient cognitive disorder, or alternatively may represent adaptive mechanisms activated for maintenance of brain functionality during aging.

## Abbreviations

AAL: Automated anatomical labeling
Aβ: Amyloid-beta
AD: Alzheimer’s disease
ApoE: Apolipoprotein E
ART: Artifact Detection Tool
BNT: Boston Naming Test
BOLD: Blood-oxygen-level dependent
DSB: Digit Span Backward
DSF: Digit Span Forward
ETH: Swiss Federal Institute of Technology
FC: Functional connectivity
FDR: False discovery rate
fMRI: Functional magnetic resonance imaging
MANOVA: Multivariate analysis of variance
MMSE: Mini-Mental State Examination
MRI: Magnetic resonance imaging
PCA: Principal component analysis
PET: Positron emission tomography
PiB: Pittsburgh Compound-B
QSM: Quantitative susceptibility mapping
SD: Standard deviation
SNR: Signal to noise ratio
T: Tesla
TE: Echo time
TMT: Trail Making Test
TR: Repetition time
VLMT: Verbal Learning and Memory Test
